# Prefrontal Neurons Encode Actions and Outcomes in Conjunction with Spatial Location in Rats Performing a Dynamic Delayed Non-Match to Position Task

**DOI:** 10.1371/journal.pone.0149019

**Published:** 2016-02-05

**Authors:** Kristen D. Onos, Miranda J. Francoeur, Benjamin A. Wormwood, Rikki L. A. Miller, Brett M. Gibson, Robert G. Mair

**Affiliations:** 1 Department of Psychology, University of New Hampshire, Durham, NH 03824, United States of America; 2 The Jackson Laboratory, 600 Main St., Bar Harbor, ME 04609, United States of America; University of Medicine & Dentistry of NJ - New Jersey Medical School, UNITED STATES

## Abstract

To respond adaptively to change organisms must utilize information about recent events and environmental context to select actions that are likely to produce favorable outcomes. We developed a dynamic delayed nonmatching to position task to study the influence of spatial context on event-related activity of medial prefrontal cortex neurons during reinforcement-guided decision-making. We found neurons with responses related to preparation, movement, lever press responses, reinforcement, and memory delays. Combined event-related and video tracking analyses revealed variability in spatial tuning of neurons with similar event-related activity. While all correlated neurons exhibited spatial tuning broadly consistent with relevant task events, for instance reinforcement-related activity concentrated in locations where reinforcement was delivered, some had elevated activity in more specific locations, for instance reinforcement-related activity in one of several locations where reinforcement was delivered. Timing analyses revealed a limited set of distinct response types with activity time-locked to critical behavioral events that represent the temporal organization of dDNMTP trials. Our results suggest that reinforcement-guided decision-making emerges from discrete populations of medial prefrontal neurons that encode information related to planned or ongoing movements and actions and anticipated or actual action-outcomes in conjunction with information about spatial context.

## Introduction

To survive in a changing world it is important to select contextually-appropriate actions that are likely to produce favorable outcomes. Medial prefrontal cortex (mPFC) is an important component of pathways that support adaptive responding. mPFC has prominent connections with sensorimotor areas of cortex and striatum that are crucial for preparing and selecting actions [1–4] and association cortices and limbic areas that represent information about recent events, spatial context, and action outcomes [5,6]. Lesions damaging mPFC affect a number of cognitive control functions that optimize adaptive, goal-directed behaviors [7,8] including delayed conditional discriminations and other tasks guided by spatial and contextual cues [9–13]. Recordings of mPFC neurons have revealed responses related to diverse aspects of reinforcement-guided decision-making, including preparation to respond, movement, actions, anticipation and delivery of reinforcement, errors, and working memory [14–30]. The coding of these different behavioral events has not been well characterized for a single task. It is not clear how response types compare, whether distinct populations of neurons represent a specific type of event-related information, or if individual neurons represent multiple types of information [31–33]. There is little known about timing differences or how neural activity representing different types of information is coordinated.

It is also not known whether or how event-related information is integrated with information about context. mPFC receives direct input from hippocampus that has been hypothesized to provide spatial and nonspatial contextual information for mPFC-dependent decision-making [19]. Tested during foraging in an open field mPFC neurons do not exhibit spatially selective responses [16,59]. Several reports have described broad spatial tuning during spatial navigation or memory tasks, however, it is not clear in these tasks whether this activity reflects spatial location per se or behavioral events that occur in consistent locations [14,17,28–30].

To examine the representation of contextual and event-related information we trained rats to perform a dynamic delayed non-match to position (dDNMTP) task designed to characterize diverse features of mPFC function using different sets of levers that are randomly selected for each trial. We analyzed neuronal activity relative to behavioral events as peri-event time histograms (PETH) and raster plots and relative to location as spatial heat maps to test whether single neurons represent both types of information. dDNMTP trials consist of a sequence of four lever presses requiring movement along equidistant pathways between levers, reinforced sample and choice responses defined by egocentric direction of movement (90° left vs. right), and an imposed delay between sample and choice phases requiring representation of sample information after the sample lever press and reinforcement are complete. Training occurred in an open arena having a diverse array of visible allocentric cues with four retractable levers and spouts for water reinforcement located 90° apart that provided multiple start locations with sample and choice levers in a consistent “T” configuration. Sample and correct choice responses are defined relative to a randomly selected base location so that locations of behavioral events varied unpredictably from one trial to the next. Rats received extensive training to assure consistent and accurate behavioral responses and sufficient trials to characterize timing properties of event-related activity.

We found a limited set of neuronal response types corresponding with specific behavioral events, with temporally-specific activity related to preparation, movement, lever presses, anticipation and delivery of reinforcement, errors, and memory delays. While many neurons responded broadly across all locations where an event occurred, some had more spatially restricted activity: responding more robustly to the conjunction of a behavioral event with a specific location. These results suggest that the capacity for adaptive decision-making arises from discrete populations of mPFC neurons that encode information related to specific behavioral events in conjunction with spatial context.

## Materials and Methods

### Ethics Statement

This research was conducted in strict accordance with the Guide for the Care and Use of Laboratory Animals of the National Institutes of Health. The protocol was approved by the IACUC at the University of New Hampshire. All surgery was carried out under anesthesia produced by the combination of ketamine and xylazine.

### Subjects

The Institutional Animal Care and Use Committee at the University of New Hampshire approved all procedures. Neuronal activity was recorded from six male Long Evans rats obtained from Harlan Laboratories (Boston, MA), weighing 350 to 500 gm during recording sessions. They were two to 9 months old at the start of training, 5 to 13 months at the start of recording, and 10 to 17 months at the end of recording. Rats were housed in plastic tubs with wood shavings on a 12:12 h light/dark cycle and tested during the light cycle. Food was provided ad libitum and water was restricted to 30 m at the start of the dark cycle on days when they received water during training and recording sessions and 60 m on days when they did not.

### Apparatus

Rats were trained and tested in a clear polycarbonate octagonal arena, 61 cm in diameter. Retractable levers (ENV-112CM, Med Associates, St. Albans, VT) were centered on four walls 90° apart (N,E,S,W), each with a stimulus light (ENV-221M, Med Associates) and drinking spout above to signal and deliver water reinforcement by activation of a miniature solenoid valve (LFAA1201518H, The Lee Co., Essex, CT). The arena was located in a Faraday cage with a screen door that provided ambient illumination and many visible external cues. A video camera was located 1.0 m above the center of the floor of the arena to record movements of rats during recording sessions. The behavioral apparatus was controlled by a PC interface (Med Associates) in an adjacent room.

Electrophysiological activity was recorded from tetrodes, through a head stage and tether (Neuralynx, Bozeman, MT) connected through either a motorized servo-controlled commutator (Neuralynx) or low torque slip-ring commutator (Dragonfly Research and Development, Inc., Ridgeley, WV) to a Neuralynx Digital Lynx SX high density electrophysiology recording system.

### Behavioral training

**Fig 1:**
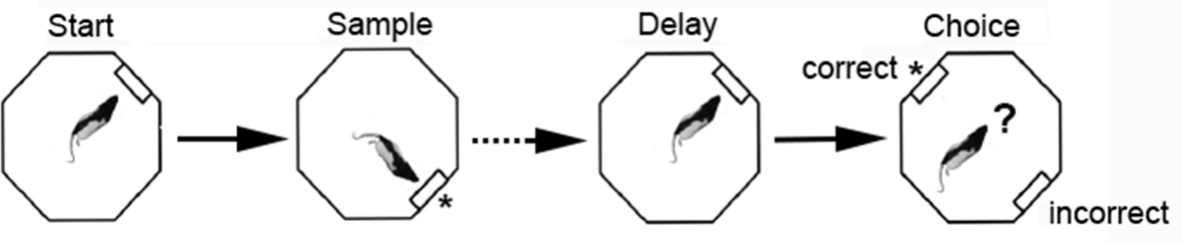
Schematic drawing of the dynamic DNMTP task. The sequence of four lever presses constituting a DNMTP trial. The base lever location where start and end of delay presses occurred was randomly selected for each trial. The sample lever was randomly selected as 90° to the left or right of the base lever. Levers 90° to the left or right of the base were extended for the choice. Reinforcement, indicated by *, was delivered to the drinking spout immediately above the lever following sample and correct (non-matching to sample) presses.

dDNMTP was trained using three levers in a “T” configuration with a central base lever extending for both start and delay responses and levers 90° to the left and right for sample and choice responses (Fig 1). The base lever location was randomly selected for each trial from either all four levers or two levers on opposite sides of the arena (see below). Thus while the “T” configuration of arms was consistent across trials, the orientation of the “T” within the arena changed unpredictably from trial to trial. Trials began with the base lever extending for the start response. The base lever then retracted when pressed causing the sample lever to extend (randomly selected as 90° to the left or right of the base). The sample lever retracted when pressed, delivering reinforcement immediately above the lever (signaled by the panel light) and causing the base lever to extend again. The base lever retracted with the first press after the memory delay ended, causing the levers 90° to the left and the right of the base to extend for the choice. Reinforcement was then delivered and all levers retracted when the rat pressed the lever not extended for the sample press (i.e. non-matching to sample position). When this was the first lever pressed during the choice phase the response was scored as correct. When rats pressed the incorrect lever first, the trial was scored as an error and rats were allowed to continue until they pressed the correct lever and received reinforcement. After a 5.0 s inter-trial interval a new randomly selected base lever was extended to begin the next trial. Reinforcement consisted of two 0.1 s (0.1 ml) pulses of water, 1.0 s apart. The memory delay was randomly selected as 1s or 5s on a trial-by-trial basis for the first four rats and was fixed at 3.0 s for all trials for the last two.

Rats were shaped and trained to perform dDNMTP to a criterion of completing 60 trials in a 60 m session with 70% correct with four possible base lever (start/delay press) locations. Rats required three to four months of daily training to reach this criterion. Tetrode arrays were surgically implanted after rats reached criterion. Following a week of recovery, water-restriction was re-instituted and rats were trained while activity was recorded. During post-surgical recording sessions, data were analyzed only for sessions where rats completed a minimum of 40 trials in a 60 m session. When rats failed to perform 40 trials with 70% correct with four possible base lever locations training was shifted to an easier version with two possible base lever locations 180° apart (N vs. S or E vs. W). Note that the structure of DNMTP trials was identical in both versions. The base levers used for the easier two location task were changed between sessions so that rats trained with N vs. S start locations one day, were trained with E vs. W start locations the next day. This was done to ensure that rats could not rely on fixed spatial cues to solve the task.

### Electrophysiological recording

Activity was recorded using an array of four tetrodes, each having four twisted 17.8 micron platinum iridium microwires (California Fine Wire, Grover Beach, CA) and contained in a stainless steel cannula. The cannula was soldered to center pin in an 18 pin Mill-Max socket with a sliver ground wire and the 16 microwires of the tetrodes attached to the other pins. Tetrode arrays were fastened through a poly(methyl methacrylate) base to a tripod of 2–56 x 15mm ss base screws that screwed into threaded sockets glued to the skull. This allowed us to lower tetrode arrays incrementally between recording sessions. Tetrodes were lowered simultaneously. Tetrode arrays were lowered by either an 1/8^th^ (0.056 mm) or a 1/16^th^ of a turn for each of the three screws following recording sessions when rats performed a minimum of 40 trials or when tetrodes had not been lowered in the previous three days.

Before being implanted each microwire electrode was tested and plated with platinum black to lower impedances to a target ≤ 200 k ohms at 1.0 kHz using a Nano-Z (Neuralynx). Recorded signals were amplified (relative to the silver ground wire inserted inside the skull) and processed using Cheetah data acquisition software. Digital signal processing low cut and high cut filters were set to 1,000 to 10,000 Hz for earlier recordings using the motorized commutator and changed to 600 to 6,000 Hz when we switched to the passive low torque commutator.

### Surgical procedures

Rats were anesthetized with an IM injection of ketamine (85 mg/kg) and xylazine (8.5 mg/kg), placed in a stereotaxic instrument, and tetrode arrays implanted using aseptic techniques 3.0 mm anterior to Bregma, 0.6 mm to the left or right of the sagittal suture. A small opening for the tetrode array was made in the skull and holes drilled for 0–80 ss skull screws to which the sockets securing the base screws of the tetrode array were attached with Grip cement (Dentsply Int., Inc., Milford, DE). Two of the rats additionally had an external cannula inserted aimed at an angle towards central thalamus for studies of the effects of thalamic inactivation on prefrontal neural activity (data not reported here). Butorphanol (0.2 mg/kg SC) was administered at the end of surgery for postsurgical analgesia.

### Histological analyses

At the completion of recording sessions, tetrode tracks were marked in four of the rats by passing 100 μV of current for 30 s using an A365 constant current stimulus isolator (WPI, Sarasota, FL). Tetrode tracks were identified without electrolytic marking in the first two animals. Rats were sacrificed three days later under deep anesthesia (100 mg/kg ketamine, 10 mg/kg xylazine IM) by transcardiac perfusion of physiological saline followed by 4% (v/v) neutral buffered formalin. Tetrodes were backed out and removed to minimize post-mortem damage and brains removed and immersed in 30% sucrose 4% neutral buffered formalin until they were ready to be sectioned. Tissue was blocked in the flat skull position using an RBM 4000C brain matrix (ASI Instruments, Inc., Warren, MI), sectioned frozen in the coronal plane at 50 μm, and stained with thionin. Tissue was examined to identify the course of the tetrode track. The locations of individual neurons recorded were inferred based on the number of turns the arrays had been advanced when neurons were recorded.

### Data analyses

**Fig 2:**
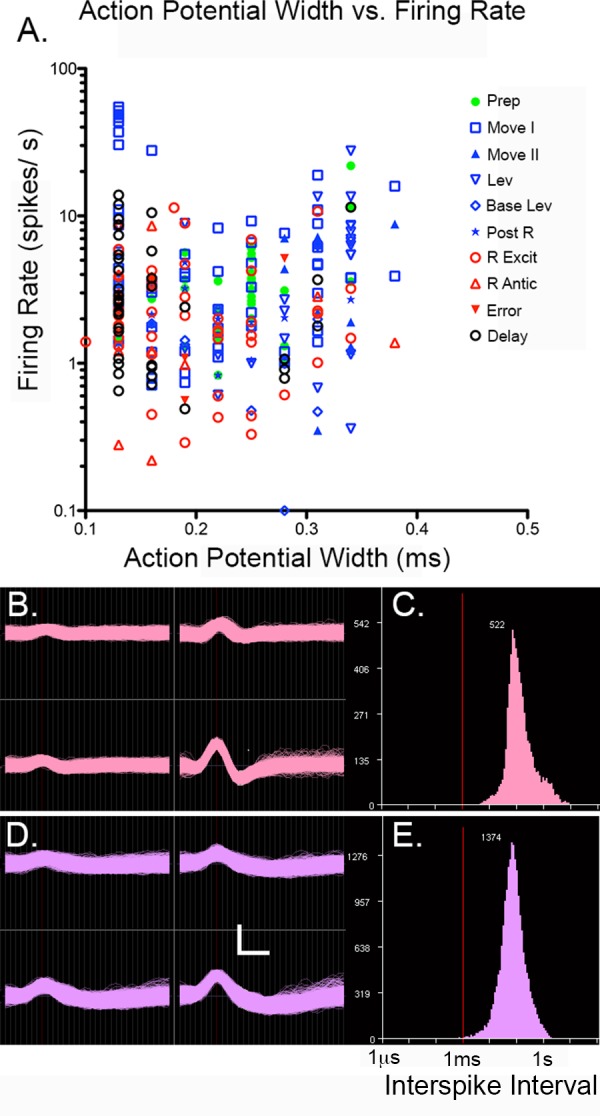
Properties of recorded action potentials. (A) Peak to trough action potential width vs. log firing rate for cells classified with each of the most commonly observed response types. (B) Tetrode recordings and (C) interspike interval (ISI) histogram from a neuron with narrow width (125 μs). (D) Tetrode recordings and (E) ISI histogram for a neuron with a wide width (344 μs). Calibration marks are 50 μV (vertical) and 200 μs (horizontal) in B and D. Red lines indicate 1 ms ISI (C, E).

Data consisted of continuous digital records of activity from each of the 16 microwire electrodes, TTL pulses marking behavioral events with specific time stamps, and HD video tracking records. Signals from microwire electrodes were processed offline using Spike Sort 3D software (Neuralynx) for automated cluster cutting to identify signals from single neurons. These plots were rotated manually to identify potential overlap between clusters. Clusters identified by automatic analyses were merged when together they constituted a well-defined cluster in 3D space and exhibited highly similar waveforms at each of the microwires in a tetrode. To make certain that we did not create a false impression of mixed coding of multiple events by inadvertently combining signals from two neurons with highly similar waveforms, we compared PETHs whenever clusters were merged. In every case where clusters were merged based on similarity of recorded waveforms, subsequent examination revealed comparable PETHs as well. The criteria for identifying isolated cells were distinct waveforms recorded by different microwires in a tetrode, a well-defined cluster in the 3-D plot, a minimum interspike interval above 1.0 ms with an interspike interval histogram peaking above 10 ms, signal to noise (peak to peak) ratio of 1.5:1, hyperpolarization that was asymmetrical with depolarization, and an L ratio <1 (Fig 2) [33]. To compare our results with previous studies we measured both peak width of action potentials using averaged waveform at the microwire with the largest response in the tetrode to define the peak (highest signal) to trough (lowest signal) and average firing rate throughout behavioral sessions by dividing total number of spikes by the length of the session.

To identify event-related activity a standard set of 13 averaged PETHs and raster plots (aligned with each of the 13 event markers recorded) were generated for all isolated neurons using NeuroExplorer (Madison, AL). These included PETHs and raster plots aligned relative to TTL pulses marking the four lever press responses, reinforcement events, and correct and incorrect choice responses. To examine potential coding over delay intervals, separate PETHs were also generated for end of delay lever presses when different directions of turning (L vs. R) or lever locations (1, 2, 3, or 4) were associated with correct (reinforced) responses. Confidence limits for PETH bins were calculated from the frequency of neuronal firing by NeuroExplorer based on the actual Poisson distribution, Prob (S = K) = exp (-C)*(C^K)/ K!, where C^K is C (the expected number of events) to the power of K. Where C≥ 30 the Gaussian approximation is used [60].

**Fig 3:**
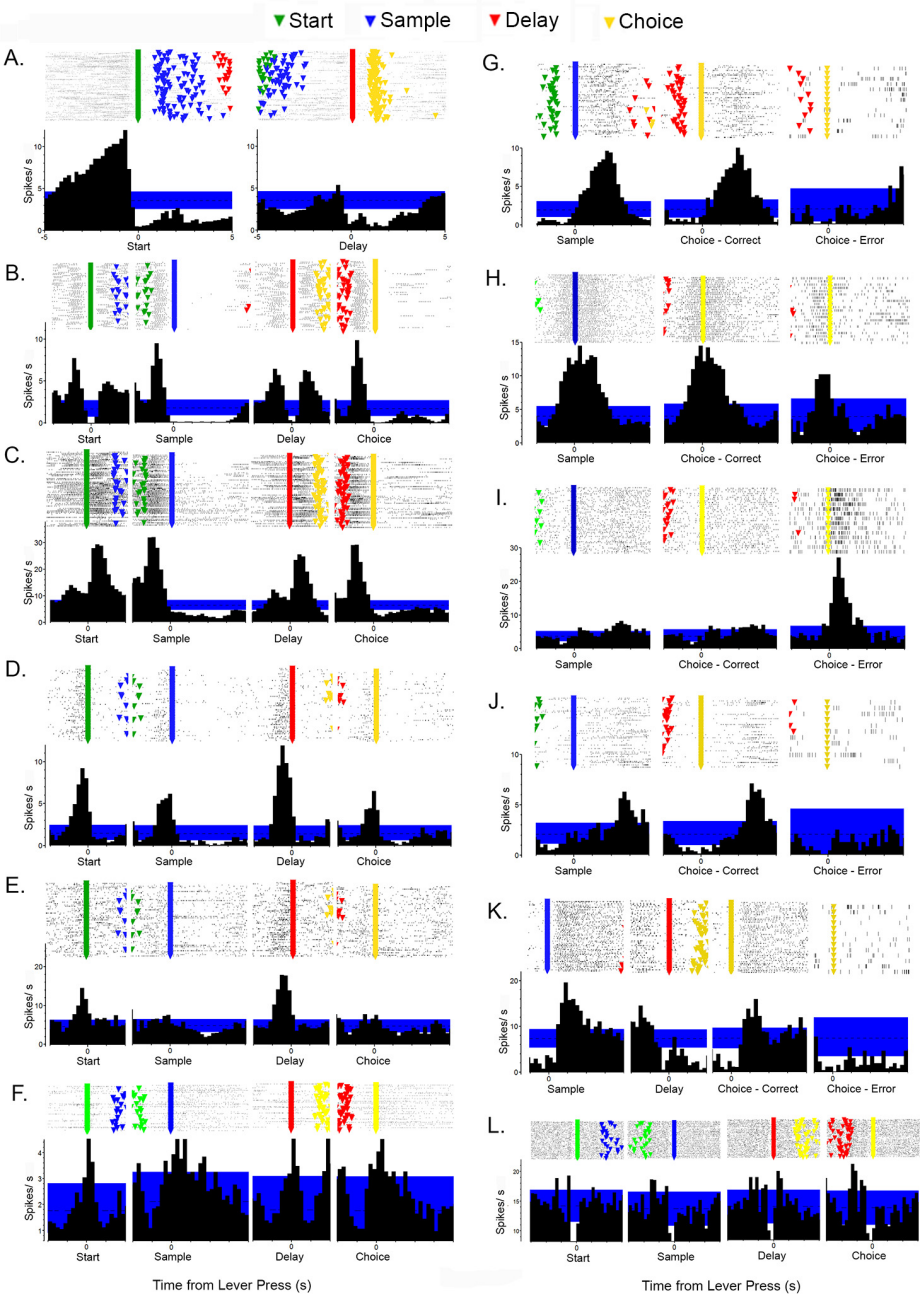
Peri-event time histograms and raster plots for different response types. Histograms show 99% confidence interval used to define event related activity in blue. Raster plots show event markers for start (green), sample (blue), delay (red), and choice (yellow) lever presses. Responses include: (A) preparatory responses relative to start and delay lever presses; (B) movement I, (C) movement II, (D) lever press excitation, and (E) base lever press responses relative to start, sample, delay, and choice lever presses; (F) lever press/ reinforcement, (G) reinforcement, (H) reinforcement anticipation, (I) error, (J) post-reinforcement responses relative to sample and correct and incorrect choice responses, (K) delay-related responses relative to sample, delay, and correct and incorrect choice responses, and (L) lever press suppression. Activity is plotted in seconds from lever presses along the abscissa.

We used two criteria to define event-related responses objectively. First, responses had to be sufficiently robust to produce PETHs with two consecutive 200 ms time bins beyond the 99% confidence interval. Second, to ensure that responses were not the result of unusual bursts of activity in a few trials, raster plots were examined to make certain that changes in activity coincident with criterion changes in PETHs occurred for at least 20% of trials examined (see Fig 3 for examples).

**Fig 4:**
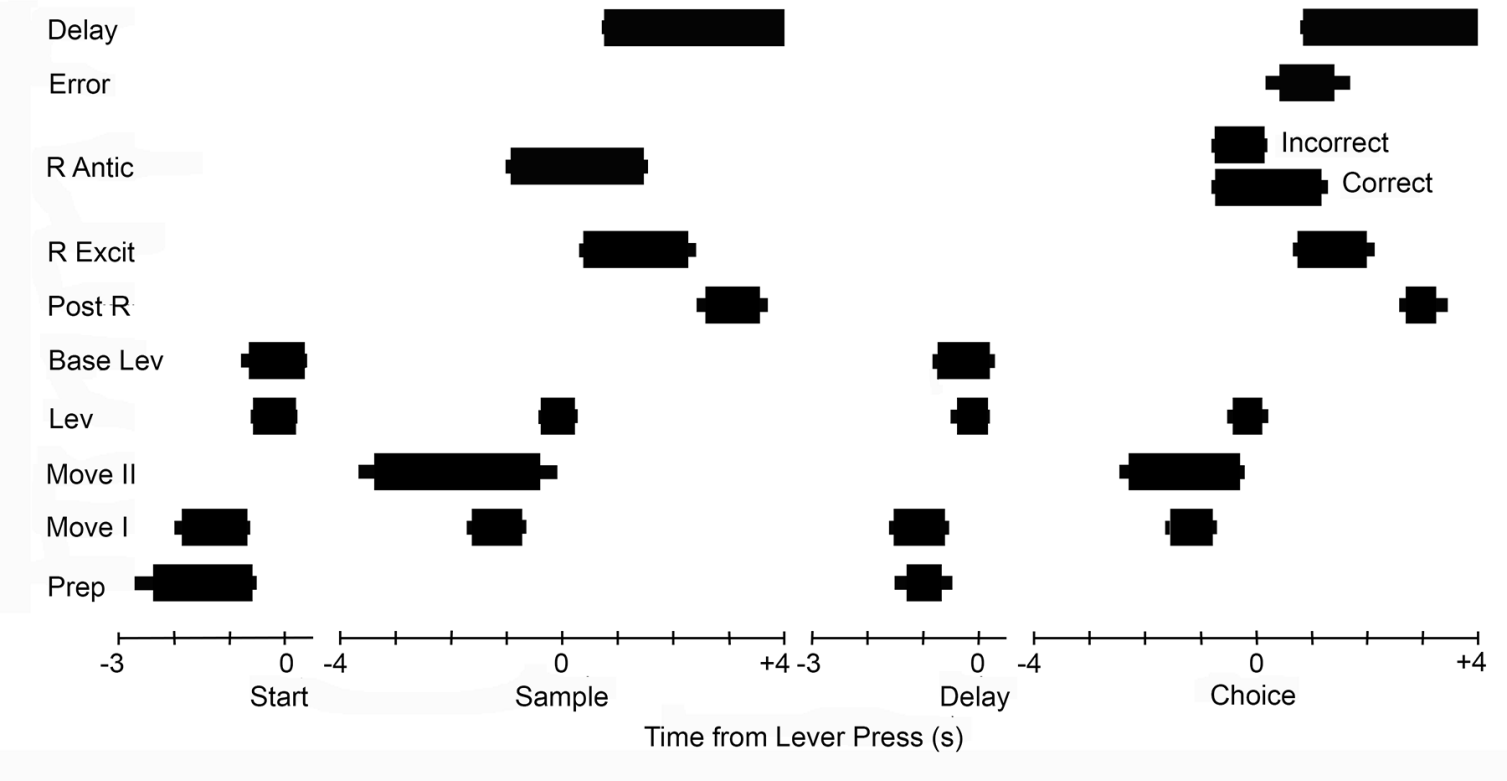
Timing of different response types during DNMTP trials. The average (thick bar) and SEM (thin bar) duration of increased activity is plotted relative to start, sample, delay, and choice lever presses. Responses were measured based on when activity was above the 99% confidence interval (see Fig 3). These analyses were restricted to responses sufficiently robust to remain consistently outside this limit during periods of elevated activity. Results are plotted for preparatory (Prep, N = 25 for start and N = 19 for delay responses), movement I (Move 1, N = 55), movement II (Move 2, N = 12), lever press (Lev, N = 24), base lever press (Base Lev, N = 13), post-reinforcement (Post R, N = 8), reinforcement excitation (R Excit, N = 31), reinforcement anticipation (R Antic, N = 17), error (N = 4), and delay (N = 19). Preparatory and movement responses are plotted relative to the lever presses they preceded. Reinforcement, error, and delay responses are plotted relative to the sample and choice lever presses that they followed.

PETH results (Fig 3) were used to quantify the timing of event-related activity in different neurons (Fig 4). These analyses focused on event-related increases in activity relative to start, sample, delay, and choice lever press responses and delivery of reinforcement. The 99% PETH confidence intervals were used to define the beginning and end of responses. The goal of these analyses was to identify and compare common patterns of activation and thus they were restricted to neurons with robust responses that remained consistently beyond the 99% confidence interval throughout periods of elevated firing. Marginal responses that fluctuated back and forth between the 99^th^ percentile during these periods were excluded from these analyses since they did not provide unambiguous indications of when responses began and ended.

Earlier studies conducted without headstage LEDs did not produce useable video tracking results. Spatial tuning analyses were conducted for all neurons with criterion event-related activity observed in later recording sessions with headstage LEDs (N = 137). In addition, spatial distribution of activity was examined for 136 of 448 neurons with comparable video tracking data that did not exhibit criterion event-related activity. This was done to determine the generality of spatial coding results. Spatial coding analyses were conducted with NeuroExplorer place cell analysis to examine the rate of cell firing in a 70 by 70 grid of bins covering the behavioral arena with a minimal time/bin of 0.2 s and minimum of 3 visits in the recording session. We used the filter on the fly in the place cell analysis to generate spatial heat maps and to plot locomotor paths for specific time intervals relative to behavioral events. To compare spatial distributions of activity, spatial response clusters were defined as groups of nine contiguous bins with at least half the maximal level of activity/ bin observed for that neuron.

## Results

Activity was recorded for a total of 344 sessions where tetrodes were turned down and rats performed a minimum of 40 DNMTP trials within 60 minutes (38 to 72 for individual animals). Rats achieved criterion of 70% correct in 269 (78%) of these (46% to 90% for individual animals). We recorded isolated activity from 900 neurons in six rats as tetrodes were advanced incrementally through mPFC. Analyses of action potential peak width and firing rate revealed peak to trough widths from 0.13 to 0.38 ms and average firing rates clustered from 1 to 10 spikes/s (Fig 2). Overall, firing rate was not correlated with peak width (r = 0.034, p = 0.581). The ranges of peak widths and firing rates overlap with signals others have attributed to somatic pyramidal and interneuron action potentials in mPFC [27,34], as well as shorter duration waveforms ascribed to axonal action potentials [35]. There was considerable overlap in these measures for neurons exhibiting different types of event-related responses (see below; Fig 2). Thus to the extent that action potential width and firing rate characterize different kinds of neurons (or signals originating from axons or soma), these results suggest that we sampled diverse types of neurons and that different event-related response types were not restricted to a particular morphological category of neuron.

PETHs meeting criteria for event-related activity were observed for 293 (32%) of the isolated neurons. Comparison of criterion responses revealed a limited set of distinct response types that accounted for 280 (96%) of behaviorally correlated cells (Fig 3). Five neurons were observed with elevated activity corresponding to both lever presses and reinforcement. No other cells were found that responded to more than one type of event. Video tracking data were available for 585 (65%) of 900 cells recorded including 137 (49%) of 280 cells with categorized response types.

### Event-related responses

**Fig 5:**
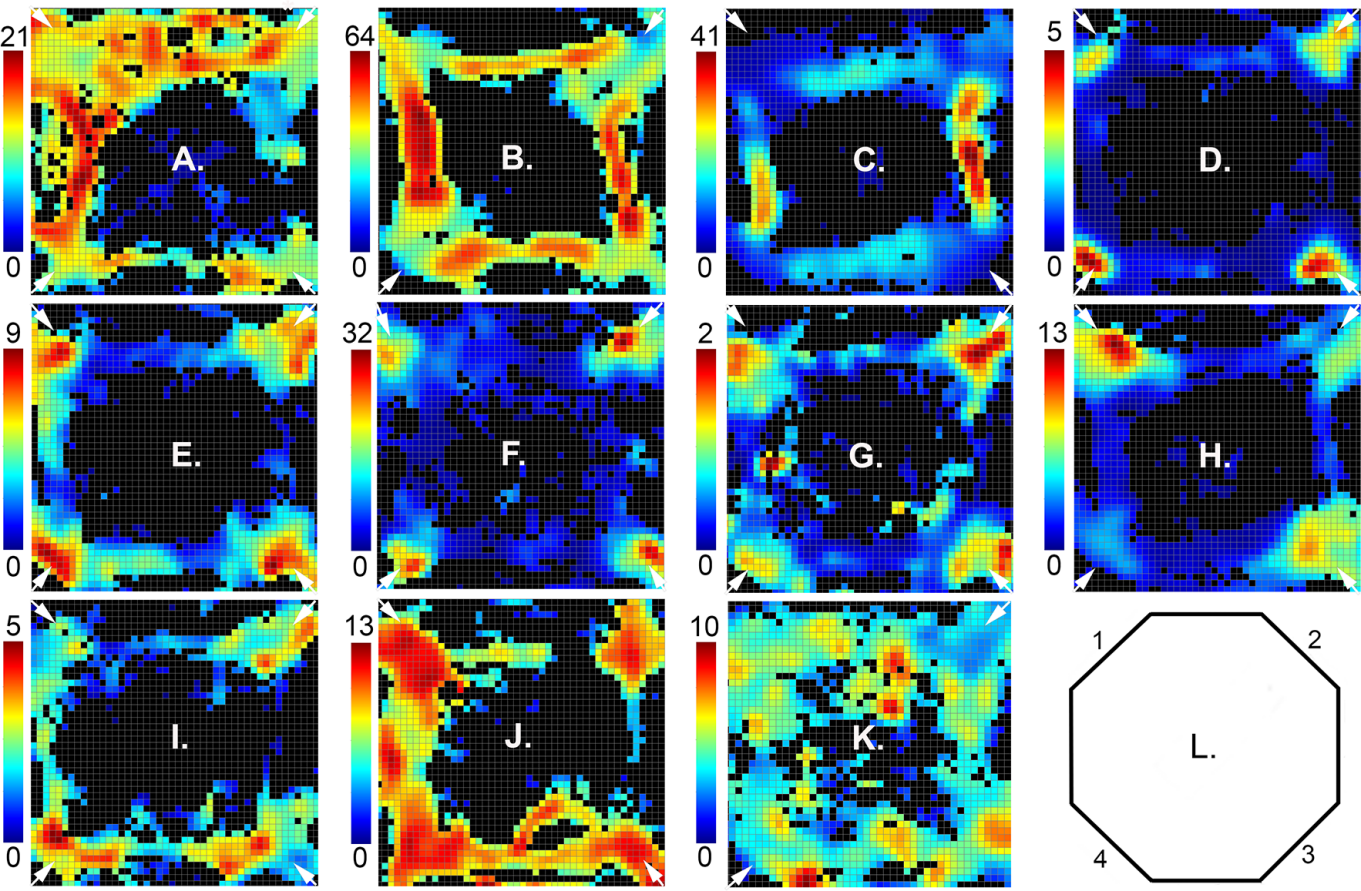
Heat maps showing spatial distribution of activity for different response types. Activity recorded for the entire session (up to 60 trials or 60 minutes) is plotted as spikes/s in each of the bins in the 70 x 70 array that met the minimum criteria of 3 visits and 0.2 s occupancy (see color scale to the left of each plot). Results are shown for: (A) preparation, (B) movement I, (C) movement II, (D) lever press, (E) base lever press, (F) reinforcement excitation, (G) reinforcement anticipation, (H) error, (I) post-reinforcement, (J) delay, and (K) reinforcement suppression. Maps are oriented with panels containing levers and drinking spouts numbered 1 to 4 centered in the corners moving clockwise from the upper left (L). The locations of base levers (either two opposite or all four) for the session depicted are indicated by small white arrows.

PETH results revealed populations of neurons with similar patterns of activity related to each of the behavioral events that comprise DNMTP trials (Fig 3). This classification of response types was supported by analyses of timing and spatial distribution of event-related activity (Figs 4 and 5). Response types included preparatory activity prior to the start of DNMTP trials, activity during periods of movement and lever press responses, anticipation and delivery of reinforcement, errors, and memory delay.

Preparatory responses (N = 33) began during the inter-trial interval and ended within 1.0s before the start lever press (Fig 3A). Smaller increases in activity were observed before delay lever presses that began the choice phase of DNMTP trials. Quantitative measurements revealed increased activity that lasted on average from 2.4 s to 0.6 s before start responses and from 1.3 to 0.7 s before delay responses for the 25 start- and 19 delay-related preparatory responses that met criterion for these analyses (Fig 4).

**Fig 6:**
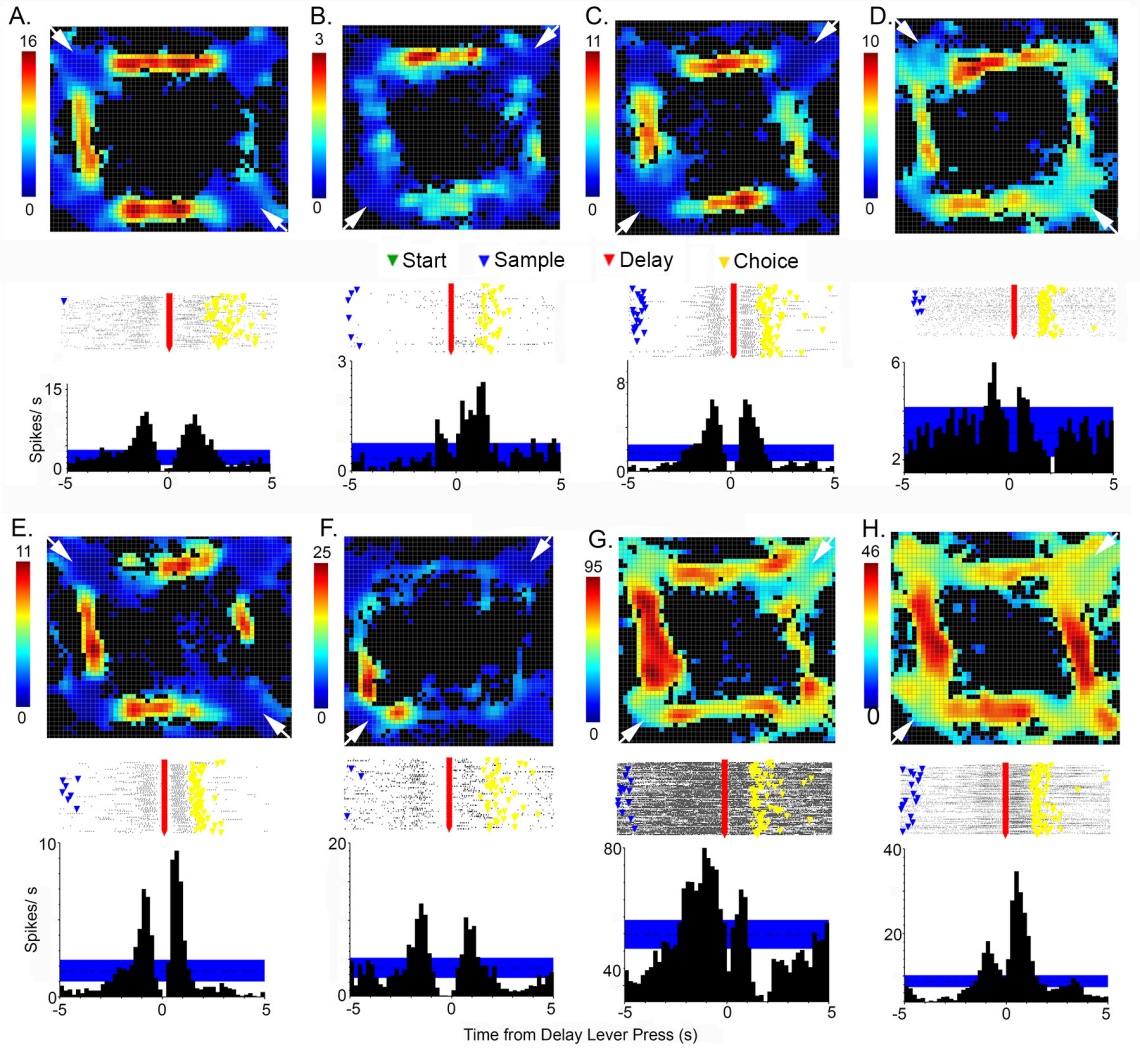
Variability in spatial heat maps, raster-plots, and peri-event time histograms (PETH) for neurons with movement I responses. PETHs and raster plots are aligned with delay lever presses to show increased activity before and after this response. The 99% confidence is indicated on PETHs in blue. Raster plots show event markers for sample (blue), delay (red), and choice (yellow) lever presses. Heat maps are oriented as in Fig 5, with activity in spikes/s indicated on the scale to the left of each plot and locations of base levers marked with small white arrows.

There were two distinct response types related to movement. Movement I responses occurred whenever rats moved towards the next lever in the DNMTP sequence (Figs 1, 3B and 6). Timing was measured for the 55 (of 68 total) responses that reached criterion for quantitative analyses. These revealed consistent periods of elevated activity for each of the four periods of movement in a trial that lasted on average 1.7s to 0.7 s before each lever press (Figs 4 and 6). One way analyses of variance (ANOVA) showed no significant differences for the onset (F_3,192_ = 2.347, p = .074) and offset (F_3,192_ = 1.062, p = .366) of movement I responses before each of the four lever presses in the sequence. Movement II responses (N = 13) occurred when rats moved from base levers towards sample or choice levers, but not when they traveled towards base levers after these responses. Quantitative analyses were based on 12 (of 13 total) responses. These revealed an earlier time of onset (3.4 vs. 2.3 s; T_20_ = 3.221, p = .004) and comparable times of offset (0.4 vs. 0.3 s; T_20_ = .514, p = .613) prior to sample compared to choice lever presses. Timing results support the distinction between movement I and movement II responses. Movement II responses lasted longer during the period preceding both sample (3.1 vs. 0.9 s; T_61_ = 7.763, p = .001) and choice (2.0 vs. 0.7 s; T_59_ = 7.217, p = .001) presses. Movement II responses began earlier than movement I before both sample (3.4 vs. 1.7 s; T_59_ = 5.519, p< .0001) and choice (2.3 vs. 1.5 s; T_58_ = 3.389, p = .001) and ended significantly later before choice (0.3 vs. 0.8 s; T_58_ = 2.039, p = .046), but not sample (0.4 vs. 0.7 s; T_59_ = 1.254, p = .215).

There were three response types associated specifically with lever press actions: excitation (N = 33) and suppressed activity (N = 5) coincident with all four presses in a trial, and excitation when the base lever was pressed (N = 13) for start or delay responses (Figs 1 and 3). Timing was measured for 24 neurons with excitatory responses to all four presses and 12 with base lever presses that reached criterion for these analyses. Cells responding to all four presses showed consistent responses that began on average 0.5 s before and lasted until 0.2 s after each of the lever press responses (Fig 4). ANOVAs revealed no significant differences for either the onset (F_3,91_ = 1.052, p = .374) or offset (F_3,91_ = 1.366, p = .118) of this activity for the four presses in a trial. Base lever responses showed similar periods of elevated activity from 0.6 s before and to 0.3 s after presses that did not differ for onset (t_24_ = .460, p = .650) or offset (t_24_ = 1.622, p = .118) for start and delay presses. There was little difference between these two types of lever press responses for the onset of activity before start (.7 vs. .6 s, t_35_ = .514, p = .610) or delay (.6 vs. .4 s; t_35_ = 1.083, p = .286) or offset of activity following start (.3 vs. .2 s; t_35_ = 2.131, p = .040) or delay (.2 vs. .1 s; t_35_ = .315, p = .755).

Reinforcement was delivered through drinking spouts and signaled by panel lights located immediately above each of the levers. It consisted of consisted of two 0.1 s pulses of water delivered 1.0 s apart. Reinforcement responses were characterized by momentarily increased (N = 42; Fig 3G) or decreased (N = 3) activity after the onset of reinforcement following sample or correct choice responses. Timing analyses based on 30 criterion excitatory responses showed increased activity from 0.6 to 2.1 s after reinforcement began. The response to sample reinforcement had an earlier onset (0.4 vs. 0.7 s after reinforcement began; T_58_ = 3.153, p = .003) than for correct choices, but ended comparably (1.0 vs. 0.8 s after reinforcement ended; T_57_ = 1.216, p = .229). Reinforcement anticipation (N = 18, with 17 sufficient for quantitative analysis) began earlier, 0.8 s before reinforcement began, and ended earlier, 0.1 s after reinforcement ended. There were no significant differences between when anticipatory responses began for sample vs. choice reinforcement (.9 vs. .7 s before reinforcement; T_32_ = 1.685, p = .102). Anticipatory responses ended later for sample or correct choice reinforcement (0.3 v. 0 s after reinforcement ended; T_32_ = 2.391, p = .023). Anticipation responses ended abruptly on average 0.1s after incorrect choices when reinforcement was not delivered (Fig 3H). Timing analyses revealed substantial differences between reinforcement and anticipatory responses. Anticipatory responses began earlier for sample (.9 s before vs. .4 s after; T_45_ = 10.061, p < .0001) and choice (.7 before after vs. .7 after; T_45_ = 11.796, p< .0001) reinforcement. Anticipatory responses also ended earlier following reinforcement for sample (1.0 vs. 0.3 s; T_45_ = 4.411, p = .001) and correct choice (0.8 vs. 0 s; T_44_ = 3.660. p = .001) reinforcement. Error responses (N = 4) were characterized by increased activity from 0.4 to 1.4 s after incorrect choices when reinforcement was not delivered (Fig 3I).

Forty neurons exhibited delay-related activity following sample responses. The most common pattern (N = 24) began with increased activity within 1.0 s of the sample response (during sample reinforcement) and decreased gradually until the delay lever press when there was a brief burst of activity ending 0.4 s after the delay response as rats moved towards a choice lever (Fig 3K). Other delay-related responses were shorter, consisting of a similarly early increase ending before the delay press (N = 9) or a later increase from 1.6 to 3.0 s after the sample press (N = 6). Comparison of PETHs aligned with delay presses for trials different S+ stimuli did not reveal any delay responses that varied as a function of the egocentric direction (L vs. R) or lever location associated with the correct choice. Delay-related responses were not tied exclusively to sample responses. Comparable responses were consistently observed following correct but not incorrect choice responses (Fig 3I). The consistent correlation of delay-related activity with reinforcement but not the direction or location of the correct choice suggests that these responses carry information about previous reward rather than cognitive aspects of spatial memory or choice.

Post-reinforcement responses (N = 8) occurred on average from 2.6 to 3.6 s after sample responses and 2.7 to 3.3 s after correct choice responses (Figs 3J and 4). This corresponds to the start of movement I responses as rats disengaged from locations where reinforcement was delivered (3.1s following sample and 2.8s following correct choice responses). Five neurons were observed that responded to the combination lever presses and reinforcement (Fig 3F). It could not be ascertained whether these represent mixed-coding of lever pressing and reinforcement or neurons anticipating reinforcement for every lever press, exhibiting a rapid drop in activity when reinforcement did not follow a lever press and sustained activity when it did. No other neurons were observed to respond to more than one type of behavioral event.

### Spatial tuning

**Fig 7:**
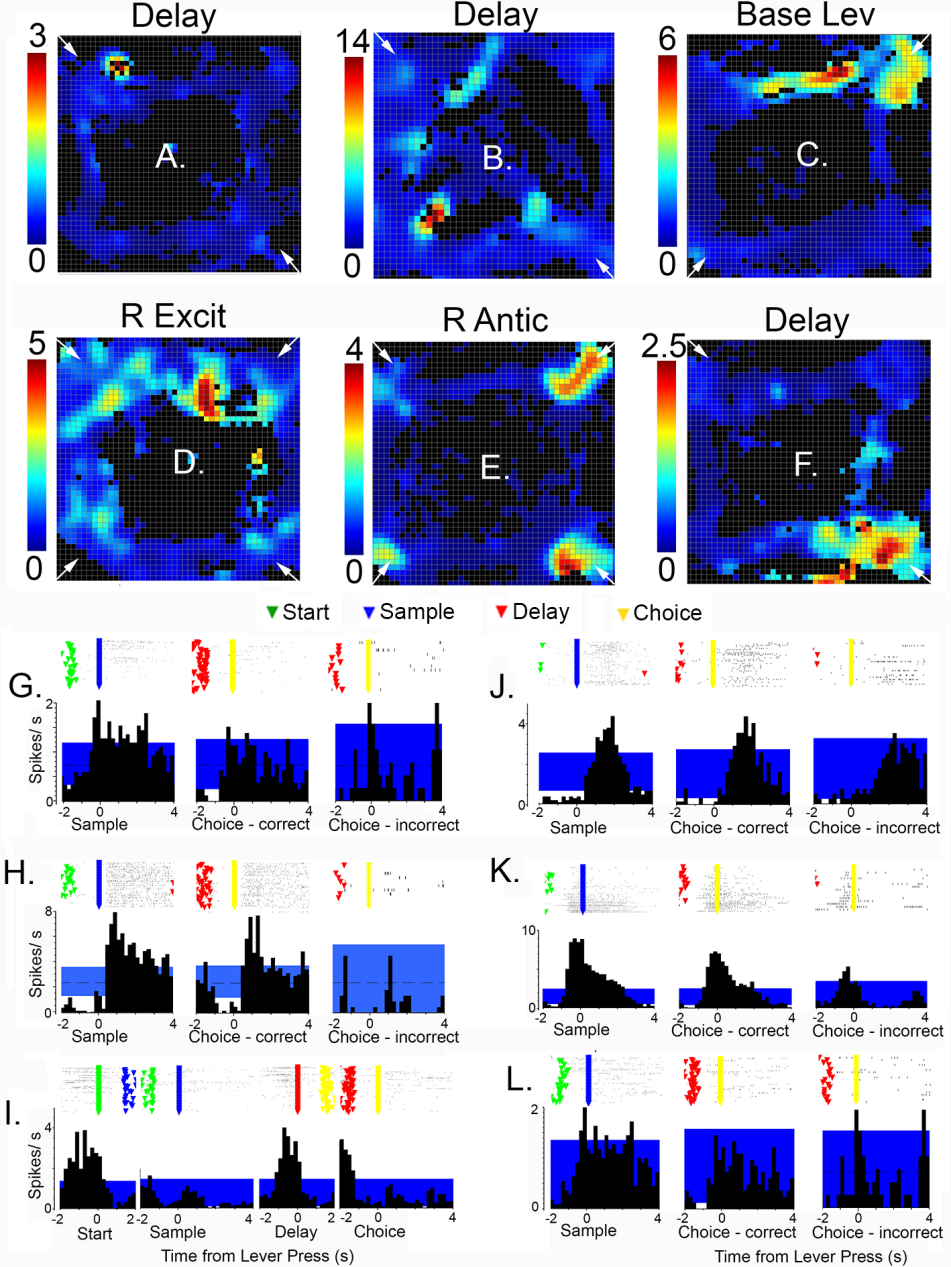
Spatial heat maps (A to F) and peri-event time histograms (PETH) and raster plots (G to L) for neurons with spatially-restricted responses. Heat maps, oriented as in Fig 5, with activity in spikes/s indicated on the scale to the left of each plot and locations of base levers marked with small white arrows. PETHs show average spikes/s relative to different lever press responses. The 99% confidence intervals are indicated by the blue areas in each PETH. Raster plots are aligned with PETHs and show event markers for start (green), sample (blue), delay (red), and choice (yellow) responses for individual trials. Results are shown for delay (A, B, F, G, H, L), base lever press (C, I), reinforcement excitation (D, J), and reinforcement anticipation (E, K) responses.

**Fig 8:**
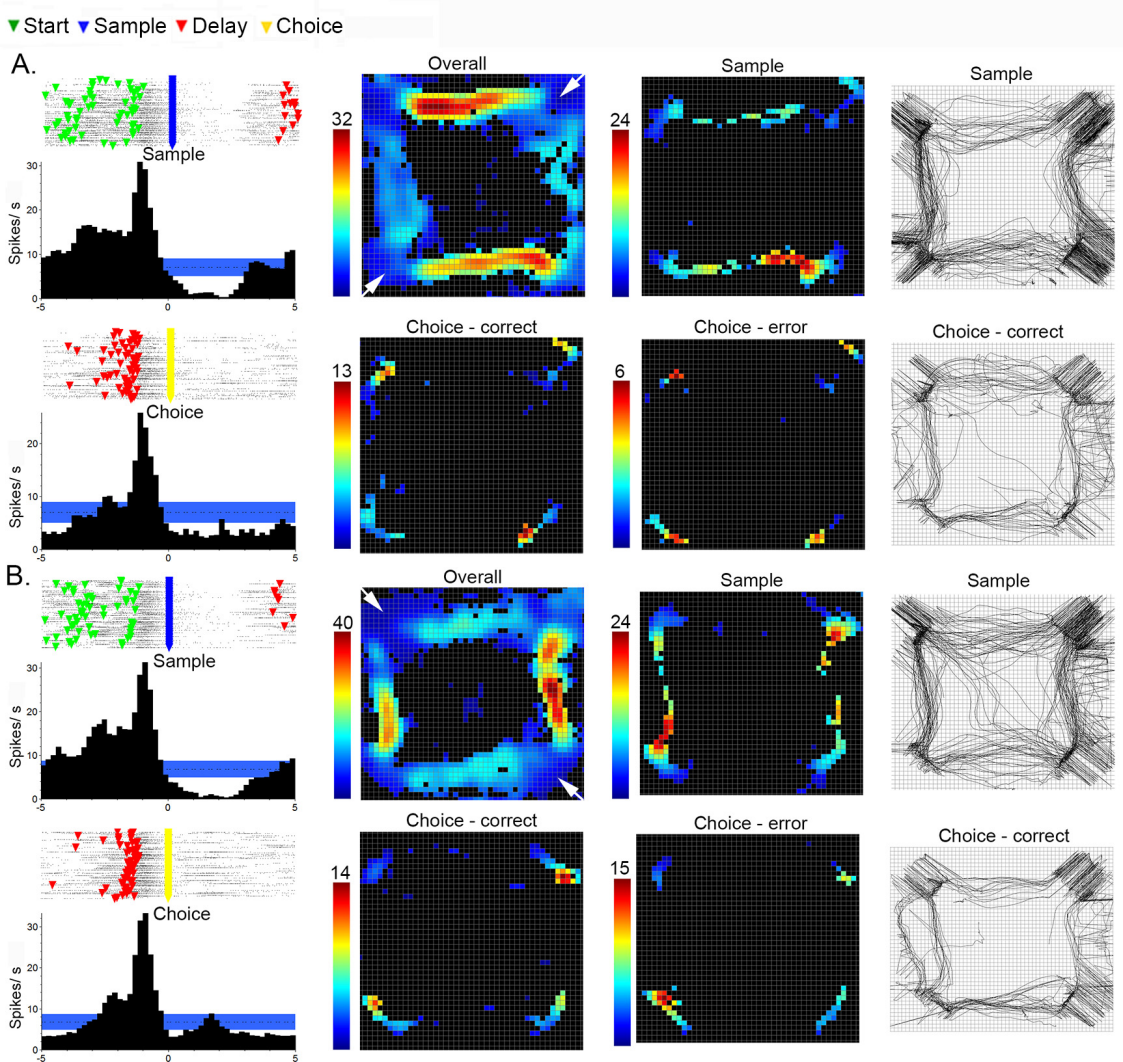
Directional coding and timing properties of movement II neurons. Raster plots and PETHs (aligned with sample and choice responses) reveal characteristic pattern of elevated activity more prolonged prior to sample than choice for two neurons (A and B). Event markers in raster plots show start (green), sample (blue), delay (red), and choice (yellow) responses in individual trials. Spatial heat maps are shown based on the entire recording session (overall) and the 4 s period preceding sample, correct choice, and incorrect choice responses. DNMTP was trained with two base lever locations for both these examples (base lever locations indicated by white arrows in corners of overall maps). Activity is plotted as spikes/ s as indicated on the scale to the left of each heat map. The paths traveled by rats during the 4.0 s preceding sample and correct choice responses are shown in the panels to the right. Both examples show activity elevated selectively to the left of the base lever location for the overall and sample plots even though the paths indicate comparable travel to the right. Activity and path maps indicate that movement II cells were active while rats visited both base and sample/choice lever locations during the 4.0 s before sample and choice responses.

Analyses of activity averaged across whole sessions revealed spatial tuning of neurons broadly consistent with event-related responses (Figs 5, 6, 7 and 8). Neurons exhibiting preparatory responses showed dispersed areas of activity that included locations of base levers consistent with elevated activity as rats moved towards base levers for the start response (Fig 5A). Movement I and II responses were associated with elevated activity along pathways between levers where rats moved between start, sample, delay, and choice responses (Figs 5B, 5C, 6 and 8).

Neurons with lever press (Fig 5D), base lever press (Fig 5E), reinforcement excitation (Fig 5F), reinforcement anticipation (Fig 5G), and error (Fig 5H) responses were associated with elevated activity in the immediate vicinity of retractable levers and water reinforcement spouts, while post-reinforcement responses tended to extend to immediately adjacent pathways connecting levers (Fig 5I). Heat maps for reinforcement suppression (Fig 5K) were characterized by more dispersed activity that was reduced where activity was decreased near reinforcement spouts. Delay-related activity was associated with elevated activity extending from sample/choice levers to base levers (Fig 5J), consistent with the timing of delay-related responses that lasted from sample reinforcement to the start of movement following the subsequent delay response.

Comparisons between neurons revealed variability in spatial heat maps for cells responding to the same behavioral event. Fig 6 shows results for eight movement I neurons, defined by elevated activity as rats moved between levers. While all exhibited elevated activity along pathways connecting levers, some exhibited elevated activity in localized areas of the arena (Fig 6B and 6F). Even those with more widely distributed activity showed distinct patterns of patchiness in the extent of activation along different portions of movement pathways (Fig 6A, 6C, 6D, 6E, 6G and 6H). The rasters and PETHs for these neurons are aligned with the delay lever press to show bursts of activity before and after the delay response, characteristic of movement I responses and distinct from movement II neurons (which do not respond before the delay response). There was also variability apparent in these measures. While some show comparable bursts of activity coming into and out of the delay response (Fig 6A, 6C and 6F), others show higher levels of activity before (Fig 6D and 6G) or after (Fig 6B, 6E and 6H) the delay press.

Spatially-restricted responses, with activity elevated in 50% or less of areas where a behavioral event occurred, were observed for 15% (20/137) of neurons with a categorized response type and video tracking data. Examples of two movement I cells meeting this criteria are shown in Fig 6B and 6F). Fig 7 shows spatially restricted examples for delay responses with activity concentrated in the area of a base lever (Fig 7A, 7G, 7F and 7L) and a sample/choice lever (Fig 7B and 7I); reinforcement excitation (Fig 7D and 7H); base lever press (Fig 7C and 7K); and reinforcement anticipation responses (with activity elevated in 2 of 4 locations associated with reinforcement; Fig 7E and 7J). Importantly, PETHs for these spatially-restricted responses are similar to neurons with more widely distributed activity. Raster plots show trial-by-trial gaps in patterns for cells with spatially-restricted coding indicative of activation during a portion of trials (compare Figs 3, 6 and 7). This pattern of activation is consistent with cells responding selectively when a behavioral event occurs within a spatially restricted field.

Movement II cells seemed to be a special case where spatially-restricted responses were more the rule than the exception. All cells for which we have video tracking data (N = 8) were trained with two-lever protocols. The other five examples were trained with the four-lever protocol. Each of these exhibited spatially-restricted fields with activation along pathways on opposite sides of the arena (Figs 5C and 8). Since base levers were in opposite corners in these sessions (1 v.3 or 2 v. 4), this pattern is indicative of directional specificity for movement to either the left or right of the base lever. To confirm this interpretation we compared spatial patterns of activity during the 4.0 s period before sample and choice responses separately. PETHs and raster plots revealed typical patterns of elevated activity before sample and choice responses, beginning earlier before the sample (see above). The overall response showed the typical pattern of activation along two opposing pathways (compare to 5C). Event-related heat maps revealed activation along these same pathways before the sample press and high levels of activity at the location of base and sample/choice levers (and drinking spouts) before sample and correct and incorrect choices. Mapping of locomotor pathways confirmed that rats traversed all pathways between levers and spent time at both base and sample/choice levers during the 4 s preceding sample and choice responses. Comparable results were observed for all movement II responses with the exception that activity along pathways between levers was not apparent in two cases. These event-related analyses confirm the directional specificity of activity during sample responses and show that movement II responses extended from when rats were at the base lever to when they were at the sample or choice levers. There were not apparent differences between activity during correct and incorrect choice responses.

**Fig 9:**
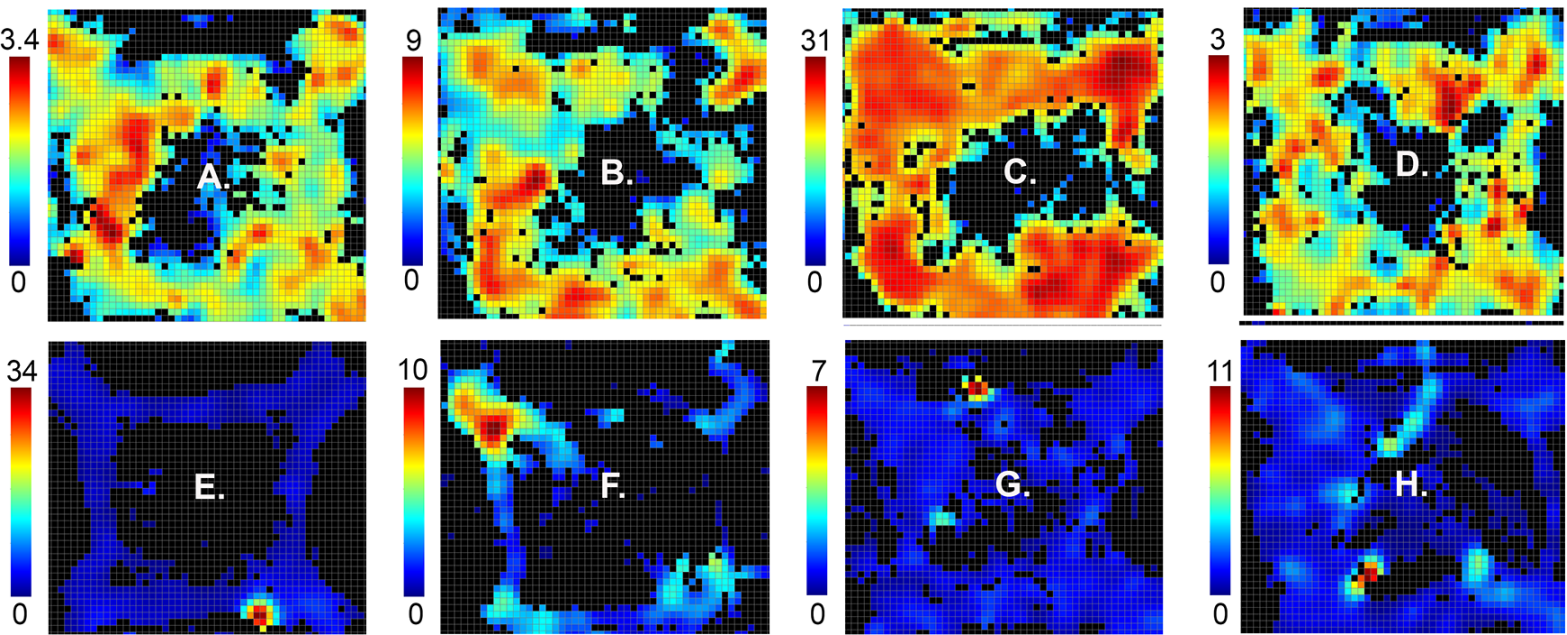
Broadly distributed and spatially-restricted firing patterns for neurons without criterion event-related responses. Spatial heat maps show the distribution of activity of neurons with broadly distributed (A to D) and restricted (E to H) spatial firing fields. Maps are oriented and neural activity is plotted as spikes/s as indicated by the scale to the left of each plot as in Figs 5–8.

Spatially restricted fields were also observed for neurons that were not associated with criterion event-related responses (34/136, 25% of cases examined). The upper panels of Fig 9A–9D) shows four examples of cells with widely distributed areas of activation that map broadly onto areas traversed by rats during DNMTP trials. Panels E to H show examples of cells with a single cluster of activity restricted to a circumscribed area of the arena. These responses were associated with comparable patterns of locomotor activity as neurons with widely distributed areas of activity. The finding of spatially restricted fields in neurons with and without criterion event-related responses suggests that there may be a population of neurons in mPFC that are specialized to represent the spatial context of behavioral events.

### Histological analyses

**Fig 10:**
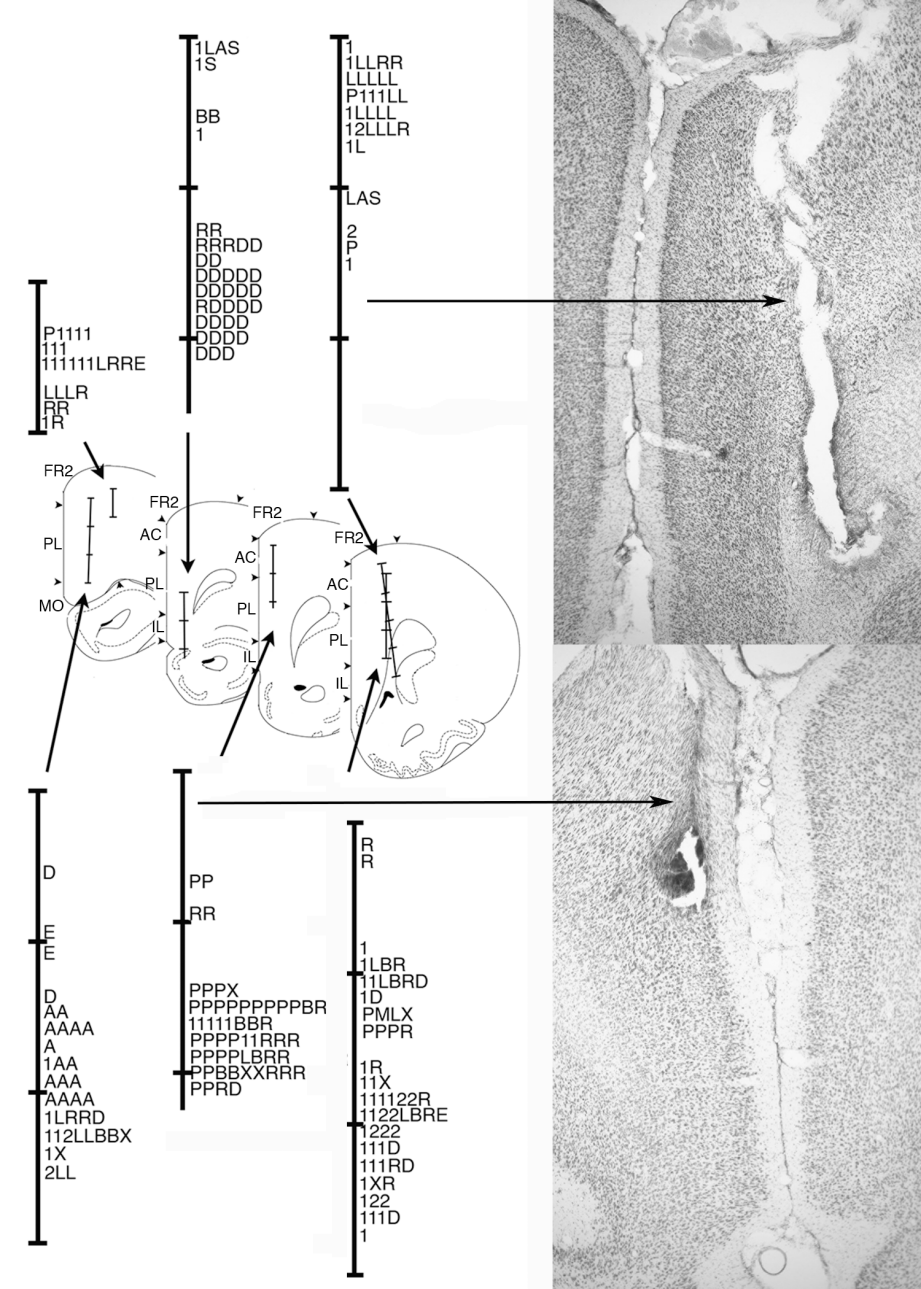
Anatomical localization of neurons exhibiting different response types. Coronal sections are modified from drawings 4.2, 3.7, 3.2, and 2.7 mm anterior to Bregma in [36]. Tetrode tracks are subdivided at intervals of 0.5 mm. Cortical fields are marked for frontal 2 (FR2), anterior cingulate (AC), prelimbic (PL), infralimbic (IL), and medial orbital (MO) areas [after 5]. Locations are plotted for neurons classed as preparatory (P), movement I (1), movement II (2), lever press (L), base lever press (B), post-reinforcement (X), reinforcement (R), reinforcement anticipation (A), error (E), and delay (D) response types. Each line represents cells recorded on two consecutive depths as electrodes were advanced ventrally. Photomicrographs show representative examples of two of the tetrode tracks (as indicated by arrows).

Histological analyses confirmed that neurons were sampled across anterior cingulate (AC), prelimbic (PL), and infralimbic (IL) areas of mPFC. Fig 10 shows the location of electrode tracks. The location of different types of neural responses are plotted along the tracks based on the number of turns the tetrode array had advanced when they were recorded. These results show considerable overlap between neurons with responses related to movement, lever press responses, and reinforcement. Preparatory responses tended to be more restricted in dorsal PL, AC, and FR2 and delay-related responses in more ventral IL and PL areas.

## Discussion

We recorded activity from mPFC neurons in rats performing a dynamic DNMTP task where locations of behavioral events varied unpredictably between trials. Individual neurons responded to distinct behavioral events, including preparation to respond, movements, lever presses, memory delay, anticipated and actual reinforcement, disengaging following reinforcement, and errors (Fig 3). Each response type was associated with consistent timing relative to behavioral events that collectively represent the temporal organization of DNMTP trials (Fig 4). Event-related responses were associated with patterns of spatial activity consistent with correlated behavioral events. Thus responses correlated with lever pressing or reinforcement were associated with activation near panels containing levers, while movement-related responses were associated with high levels of activity along pathways connecting these panels (Figs 5–7). There was substantial variability in spatial tuning of neurons with similar event-related responses, including a subset with more spatially restricted fields representing the coincidence of behavioral events and circumscribed locations, for instance lever presses at a specific lever location or movement over a limited segment of pathways between levers (Figs 6 and 7). These results suggest that the capacity for reinforcement-guided decision-making emerges from discrete populations of mPFC neurons with precisely-timed responses that encode information related to planning, movement, actions, and outcomes in conjunction with spatial location.

### Preparation, movement, and lever press responses

Reinforcement-guided decision-making requires organisms to plan and execute actions to achieve an intended outcome [1,4,37]. Here we observed neuronal responses consistent with each of these functions. Preparatory neurons fired prior to start and delay lever presses that marked the beginning of sample and choice phases of DNMTP. Movement-related responses were observed that both linked successive lever press responses and defined the direction of reinforced sample and choice responses. Lever press neurons were most active either during all four presses in the sequence or selectively when the base lever was pressed to begin the sample or choice phase.

Voluntary movements are planned before they are executed. The early offset of preparatory responses is consistent with a planning function related to the sequence of actions that constitute DNMTP trials or preparation of potential choice responses in advance of decision-making [1,4,37]. Jung, et al. [14] observed rat mPFC neurons that were active prior to the start of trials in an 8-arm radial maze task. Totah, et al. [21,25] report preparatory responses of rat PL and AC neurons during a visual attention task related to both pre-stimulus sensory attention and action preparation. Our results agree with Totah, et al. [21,25] in finding neurons with preparatory responses in more dorsal areas of mPFC (Fig 10) that have strong connections with sensorimotor areas of cortex and thalamus: an organization that seems consistent with top down control of motor function [5,6]. Lesions here are associated with a selective increase in the time taken for rats to initiate learned action sequences [38]. This association is consistent with evidence implicating human cingulate and pre-supplementary motor cortex in preparation for self-initiated movements [39,40].

Movements in DNMTP link the learned sequence of lever presses that constitute trials and define sample and choice responses. Movement I activity occurred during each movement between lever press responses in DNMTP along pathways connecting levers. The levers extended for start, sample, delay, and choice responses required the same distance of travel. Thus the uniform timing of movement I responses (Figs 4 and 7) seems consistent with a simple locomotor function. Movement II responses appear more complex. These occurred during movements towards sample and choice levers from the base lever, but not during the return from sample to delay or prior to start lever press responses. They started earlier and lasted longer than movement I responses, began earlier prior to sample than choice responses, and showed evidence of directional specificity related direction of travel (L vs. R) from the base lever. These features seem consistent with a decisional process, coding direction of movement during exploratory (sample) and decision-making (choice) activity.

Simple lever press responses were active an average of 0.7 s beginning 0.5 s before the lever switch closure that defines lever press responses. Base lever press responses were active for a similar period of time during start and delay lever presses that began the sample and choice phases of DNMTP. Their absence during sample and choice lever presses suggest that they signal information about the organization of DNMTP trials and not simply the actions involved in the lever press response. Post-reinforcement responses occurred from 1.0 to 2.5 s after reinforcement ended, coincident with the end of reinforcement-related responses and the start of movement I activity. Their activity was elevated at the site of reinforcement and for a limited distance from along pathways connecting levers. This timing corresponds to when rats disengage from reinforcement spouts, marking the end of a sample or choice phase and the start of movement towards the next lever in the DNMTP sequence.

Previous studies have revealed mPFC responses related to movement [14,19] and to lever press and nose poke responses [15,23,24,26]. Here we found a distinction between cells that respond whenever a common action occurs (movement I, lever press) and others with responses that reflect the organization of DNMTP trials (movement II, base lever press, post-reinforcement). The discovery of neurons responding to discrete actions and others to the organization of action sequences seems consistent with evidence that mPFC plays a critical role in chunking actions into organized sequences [38,41,42].

### Action outcome and delay responses

Flexible decision-making, where different choices are reinforced on different trials, requires rats to anticipate the likely consequences of a choice and to monitor the outcome. Multiple reports indicate that PFC neurons signal both expectation and delivery of reinforcement and unreinforced errors in humans, monkeys, and rats [20,29,43,44]. Here anticipatory responses began 0.8 s before sample and choice responses that delivered reinforcement, coincident with the movement-related activity as rats traveled towards sample and choice response locations, and ended 0.1 s after reinforcement ended (Figs 3 and 4). Anticipatory responses also ended abruptly 0.1 s following an incorrect choice lever press, providing immediate feedback when expected reinforcement was not delivered. Reinforcement responses began significantly later, on average 0.6 after reinforcement began and lasted 1.5 s, until 0.9 s after reinforcement ended. This timing seems more consistent with the consumption of reinforcement than with the panel light signaling reinforcement (on throughout the 1.2s reinforcement event) or delivery of reinforcement (two 0.1s pulses of water at the beginning and end of the reinforcement event). Error responses occurred from 0.4 to 1.4 s after incorrect choices when reinforcement was not delivered. In addition, delay-related responses were consistently correlated with preceding reinforcement following with sample and correct choice responses.

PFC neurons in primates and rodents exhibit sustained excitatory activity during delay periods thought to represent information that allows organisms to select correct responses in working memory tasks [18,45–50]. Here, prolonged periods of activity were observed following reinforced sample responses that were not related to either the direction or location of correct choice responses. The finding of increased activity in these neurons following correct (reinforced) but not incorrect choices suggests that these responses are more concerned with previous reinforcement than cognitive aspects of spatial memory. Horst and Laubach [23] described a similar population of mPFC responses related to outcomes of preceding trials in spatial delayed alternation. While it is unclear whether these responses carry information predicting correct choices, they do not rule out the possibility. Delay-related responses lasted from reinforcement at the sample location until after the delay response, bridging the critical period from the sample until movements are initiated towards a choice lever. At least some delay-related responses carry information about the spatial context (Fig 7) representing a potential solution for selecting a correct choice. Lesion studies have implicated subregions of mPFC with working memory for both egocentric responses and allocentric location [51].

The number of neurons with reinforcement-related responses is indicative of the importance of feedback about action outcomes for the diverse functions mediated by these areas [7,8]. The finding of responses anticipating reinforcement seems consistent with lesion studies that have implicated mPFC with the ability to select actions based on their likely outcome in studies of outcome devaluation [52], degraded contingency between instrumental actions and reward [53], and conflicts produced by incongruent action-outcome associations [54]. The finding of prolonged responses following reinforcement for sample and correct choice responses suggest that information about preceding reinforcement guides decision-making in DNMTP.

### Representation of spatial context

By varying the base lever location unpredictably between trials the dynamic DNMTP task provides a means to compare responses to common behavioral events in different spatial locations. Previous studies have described broad spatial tuning of mPFC neurons, however, these have been observed in tasks that confound spatial location with behavioral events that occur in a consistent place [14,17,28–30]. Our results revealed substantial variability in spatial patterns of activity in neurons with similar event-related activity, including some restricted to a circumscribed area of the behavioral arena (Figs 6 and 7). These neurons exhibited averaged PETHs consistent with other neurons exhibiting the same response type and raster plots indicating activation during a subset of trials—presumably those where the behavioral event corresponded with the spatial field (compare Figs 3 with 6 and 7). These results seem consistent with population encoding of spatial location, one carrying information about the timing of behavioral events as well as the location where they occur.

In addition all movement II responses examined with two start locations exhibited activation for two (of four) paths traversed (Figs 5C and 8). These movement II spatial fields were always at opposite sides of the arena, a pattern indicative of activation during a specific direction of travel (L or R) from the base levers (which were always 180° apart during training with two base lever locations). Spatial analyses restricted to the period of elevated movement II activity (4.0 s before sample and choice responses) confirmed directional responding during the sample phase and showed that movement II neurons fire from when rats are at the base lever until they reach the lever where sample or choice levers are pressed.

To examine the generality of spatially-restricted fields, spatial heat maps were examined for the 136 neurons with video tracking data that did not exhibit criterion event-related responses. Most (75%) showed widely distributed activity through all areas crossed during DNMTP responding (Fig 9A–9D). Others exhibited spatially restricted fields that encompassed a limited area (<10%) traversed during DNMTP responding (Fig 9E–9H). Locomotor activity during DNMTP did not represent a uniform sampling of floor space and thus these maps cannot be compared directly with place fields observed in random foraging tasks [55]. It should be noted that the lack of a criterion event-related response does not indicate that these neurons did not exhibit event-related activity. It is possible that they responded to events that did not correspond with the behavioral markers used to generate PETHs or that their spatial fields were so restricted that they did not produce criterion PETH responses when activity was averaged across all trials and locations.

mPFC participates in neural networks that represent contextual information about location and prior events involving medial temporal lobe and related cortical areas [22,56–58]. It is not known how PFC neurons encode contextual information. Place-related activity has been observed in monkey PFC during spatial working memory tasks [48]. Hyman, et al. [24] report that the activity of mPFC ensembles changes when rats are moved between environmental contexts. Hok et al. [17] report that about a quarter of mPFC neurons exhibit place-specific activity during goal-oriented navigation that is not observed during simple foraging [16,59]. Here a similar proportion exhibited spatially-restricted activity during DNMTP trained in an open arena. Some of these appear to be action/outcome maps, representing the conjunction of behavioral events and spatial location (Figs 6–8). Others did not exhibit criterion event-related activity (Fig 9), although it is possible that their activity corresponded to behavioral events that did not give rise to criterion PETHs. The conjunction of activity related to events and location confirms the importance of mPFC as a downstream target of spatial processing systems in medial temporal lobe—one that maps data about spatial location onto information about forthcoming and ongoing actions and action outcomes in neural networks that give rise to reinforcement-guided decision-making.
